# The Heredity of Vice

**Published:** 1913-05

**Authors:** 


### The Heredity of Vice.

The most important fact brought out by the investigations of the recent vice commissions is that immorality is an inherent characteristic. Either the predisposition for its development under suitable environment or the fully developed article itself is inborn. The moral pervert exists in spite of his environment, because of this hereditary element. Mental or physical characters cannot be formed by spontaneous generation any more than gold can be transmuted from the baser metals. They are the resultant of the germinal cells that effect the birth of the individual. Environment may hold in check and rescue the less resistant characters, but it cannot eradicate an inherent element. This will continue to reappear in the succeeding generations, unless checkmated by natural selection. Therefore, to attempt to reform a people by improving the environment alone or having nothing more than public sentiment to garrison the actions of the individual is a fruitless task—and more lamentable because of the distressing havoc that is wrought. Such national house cleaning has its advantage in purifying the atmosphere for a while, but when the storm clears away, the dust and mire of immoral pollution settles back again, emitting as much stench as ever.

So long as the game is popular, so long as we have leaders with the sort of quality necessary to keep up the agitation and the particular magnetic attraction that will abstract the coin necessary to keep the movement in the limelight, there will be the appearance of reform. At least, vice will not be so obtrusive, and there will be a greater play of pretended morality. Like the waves of religious excitement that periodically sweep over the nation, these moral awakenings appear as a psychological phenomenon, to as quickly subside when the fire of enthusiasm burns out.

The task of effecting permanent results is the work of generations. Man was not made in a day. He is what his ancestors have evolved for him, and he stands as a link in the chain of heredity, to transmit his inherited characteristics on to the next generation. That which affects the germinal cells of the body can alone modify the quality of these characters. Overworked mothers, bad nutrition, excesses in alcohol, syphilis, tuberculosis, the strife and competition of our present civilization can so modify these cells, and their influence is manifested in the next generation by a less stable nervous system and resisting power. These weaknesses, unless neutralized by the introduction of stronger characters, appear in the following generation as neurasthenia and loss of efficiency, which, in the succeeding generation, is responsible for many of the insane, imbeciles, idiots, epileptics and feeble-minded.

The converse of the proposition is likewise true. By prohibiting the propagation of undesirable characters they would soon become eliminated. Build upon this basis strong manhood and womanhood. Encourage the selection of the characters of virtue, strength, courage, patriotism, fidelity and unselfishness. When these are obtained as the controlling influence in society, moral reform and virtue will be a reality.

